# Commercial Honeybush (*Cyclopia* spp.) Tea Extract Inhibits Osteoclast Formation and Bone Resorption in RAW264.7 Murine Macrophages—An *in vitro* Study

**DOI:** 10.3390/ijerph121113779

**Published:** 2015-10-28

**Authors:** Amcois Visagie, Abe Kasonga, Vishwa Deepak, Shaakirah Moosa, Sumari Marais, Marlena C. Kruger, Magdalena Coetzee

**Affiliations:** 1 Department of Physiology, Faculty of Health Sciences, University of Pretoria, Pretoria 0001, South Africa; E-Mails: amcois.visagie@gmail.com (A.V.); abe.kasonga@up.ac.za (A.K.); viishwadeepak@gmail.com (V.D.); shaakirah.moosa@up.ac.za (S.M.); sumari.marais@up.ac.za (S.M.); 2 School of Food and Nutrition, Massey Institute for Food Science and Technology, Massey University, Palmerston North 4442, New Zealand; E-Mail: m.c.kruger@massey.ac.nz; 3 Department of Human Nutrition, Faculty of Health Sciences, University of Pretoria, Pretoria 0001, South Africa; 4 Institute for Food, Nutrition and Well-being, University of Pretoria, Pretoria 0001, South Africa

**Keywords:** honeybush tea, osteoclast, bone resorption, RANKL, RAW264.7 murine macrophages

## Abstract

Honeybush tea, a sweet tasting caffeine-free tea that is indigenous to South Africa, is rich in bioactive compounds that may have beneficial health effects. Bone remodeling is a physiological process that involves the synthesis of bone matrix by osteoblasts and resorption of bone by osteoclasts. When resorption exceeds formation, bone remodeling can be disrupted resulting in bone diseases such as osteoporosis. Osteoclasts are multinucleated cells derived from hematopoietic precursors of monocytic lineage. These precursors fuse and differentiate into mature osteoclasts in the presence of receptor activator of NF-kB ligand (RANKL), produced by osteoblasts. In this study, the *in vitro* effects of an aqueous extract of fermented honeybush tea were examined on osteoclast formation and bone resorption in RAW264.7 murine macrophages. We found that commercial honeybush tea extract inhibited osteoclast formation and TRAP activity which was accompanied by reduced bone resorption and disruption of characteristic cytoskeletal elements of mature osteoclasts without cytotoxicity. Furthermore, honeybush tea extract decreased expression of key osteoclast specific genes, matrix metalloproteinase-9 (MMP-9), tartrate resistant acid phosphatase (TRAP) and cathepsin K. This study demonstrates for the first time that honeybush tea may have potential anti-osteoclastogenic effects and therefore should be further explored for its beneficial effects on bone.

## 1. Introduction

Bone remodeling is a physiological process that involves the resorption of microscopic amounts of bone tissue by osteoclasts, followed by the formation of new bone at the same location by osteoblasts. When bone remodeling is disrupted in a way that the rate of bone resorption exceeds the rate of bone formation, progressive bone loss occurs which can result in diseases such as osteoporosis [1]. Osteoclasts are large, multinucleated cells derived from the fusion of hematopoietic mononuclear precursors of monocytic lineage. Osteoblasts secrete the soluble factors, receptor activator of nuclear factor kappa-β ligand (RANKL) and macrophage colony stimulating factor (M-CSF), to stimulate osteoclast differentiation and fusion of osteoclast precursors [2]. During the differentiation process, the actin cytoskeleton forms a distinctive ring at the sites of bone contact to aid in attachment and maintain the structural integrity of the osteoclast [2,3]. At this stage, mature osteoclasts secrete tartrate-resistant acid phosphatase (TRAP) which aids in the degradation of the bone matrix [4]. Furthermore, metalloproteinases (MMP-9) and cathepsin K are released by mature osteoclasts to degrade bone by removing bone-lining collagen [5,6,7,8].

Honeybush (*Cyclopia spp.)* is a naturally sweet tasting, caffeine free tea indigenous to the Eastern and Western Cape regions of South Africa. Its natural sweetness and pleasant honey aroma have made honeybush a popular herbal tea in South Africa, with growing interest worldwide [9]. *Cyclopia* species are native to the fynbos biome and arise from the coastal plains and mountain areas [9]. There are at least 23 known species of *Cyclopia* [9]. Six of these *Cyclopia* species, *C. subternata, C. sessiflora*, *C. longifolia, C. maculata, C. genistoides* and *C. intermedia,* are of commercial importance for their use in honeybush teas. *C. intermedia*, commonly known as “Bergtee”, is the primary source of commercial honeybush herbal tea, and is frequently combined with *C. subternata* and *C. sessiliflora* during production of the tea [10,11]. During processing, unfermented honeybush is oxidized to produce fermented tea, which has the distinctively sweet aroma and dark red-brown colour of honeybush tea [9]. Aqueous extracts from both fermented and unfermented honeybush are rich in polyphenolic compounds and have shown anti-inflammatory, antioxidant and anti-mutagenic properties, however its anti-osteoclastogenic potential remains unexplored [10,11,12,13]. Encouraged by the rich polyphenolic content of honeybush, as well as the bone protective effects reported in other tea extracts rich in polyphenols [14,15], we sought to determine whether commercial honeybush tea extract can exert bone protective effects by altering osteoclast formation and activity. In this study, the effect of aqueous extract of fermented honeybush tea was examined on RANKL-induced osteoclast formation and bone resorption in RAW264.7 macrophages.

## 2. Experimental Section

### 2.1. Reagents and Materials

All chemicals of analytical grade were obtained from Sigma Chemical Company (St. Louis, MO, USA). Dulbecco’s modified eagle medium (DMEM) and fetal bovine serum (FBS) were provided by GIBCO (Grand Island, NY, USA) and Amersham (Little Chalfont, UK), respectively. Honeybush tea (*Cyclopia intermedia*) was sourced from Vital Health Foods (Kuils River, South Africa). Cover slips and cell culture plates were provided by LASEC (Cape Town, South Africa). RANKL was purchased from Research and Diagnostic Systems (R & D Systems, Minneapolis, MN, USA). Leukocyte Acid Phosphatase (TRAP) kit (#387A-KT) was obtained from Sigma-Aldrich Inc. (St. Louis, MO, USA). Alamar blue reagent and Alexa Fluor 568 phalloidin probe were obtained from Life Technologies (Carlsbad, CA, USA). Corning osteoassay plates were supplied by Corning Incorporated (Corning, NY, USA). M-MuLV reverse transcriptase was purchased from New England Biolabs (Hitchin, UK). Roche FastStart Essential DNA Green Master was purchased from Roche (Basel, Switzerland).

### 2.2. Tea Extract Preparation

A stock concentration of 125 mg·mL^−1^ of honeybush tea extract was prepared in deionized boiled water in a 50 mL conical tube [16]. The tea extract was allowed to steep for 30 min while mixing slowly with a Stuart rotator mixer (LASEC) at 30 rpms for 60 min at room temperature. Thereafter the tea was filtered using Whatman No 1 filter paper followed by a 0.22 µm sterile syringe filter. Aliquots were stored in the dark at −20 °C until further use. The highest concentration of water did not exceed 0.8% in the experiments and this concentration was used for the vehicle control.

### 2.3. Analysis of Known Flavonoids by HPLC-DAD

Major phenolic compounds in the tea extract were quantified at the Post-Harvest and Wine Technology Division, Agricultural Research Council (ARC) Infruitec-Nietvoorbij (Stellenbosch, South Africa) by high performance liquid chromatography with a photodiode array detector (HPLC-DAD) as previously described [17].

### 2.4. Cell Culture and Maintenance

RAW264.7 murine macrophages (#TIB-71) were obtained from the American Type Culture Collection (ATCC, Rockville, MD, USA). Cells were maintained in DMEM supplemented with 10% heat-inactivated FBS and antibiotics: streptomycin (100 μg·mL^−1^), fungizone (0.25 μg·mL^−1^) and penicillin (100 µg·mL^−1^). Cells were incubated at 37 °C in a humidified atmosphere with 95% air and 5% CO_2_.

### 2.5. Alamar Blue Assay

RAW264.7 macrophages were seeded into a 96-well culture plate at a density of 5 × 10^3^ cells/well, in DMEM containing 10% heat-inactivated FBS and antibiotics. After incubation overnight for attachment, media was changed and the cells were exposed to increasing concentrations of honeybush tea extract (62.5–1000 µg·mL^−1^). Cells were further incubated for 48 h. Alamar blue assay was conducted according to the manufacturer’s instructions and fluorescence was measured at 570 nm and 600 nm on a microplate reader (BioTek Instruments Inc., Winooski, VT, USA). Results were expressed as a percentage relative to the control.

### 2.6. Investigation of Osteoclast Formation and Activity

RAW264.7 cells were seeded into a 24-well plate at 2 × 10^4^ cells/well in DMEM containing 10% heat-inactivated FBS in the presence of 15 ng·mL^−1^ RANKL and increasing concentrations of honeybush tea extract (62.5–1000 µg·mL^−1^) for 5 days. Medium and all factors were replaced on day 3.

#### 2.6.1. Cell Morphology: PlasDIC

At the end of culture, polarization-optical transmitted light differential interference contrast (PlasDIC) pictures were taken using a Zeiss inverted Axiovert CFL40 microscope, containing a specialized PlasDIC filter, with a Zeiss Axiovert MRm monochrome camera (Carl Zeiss AG, Oberkochen, Germany).

#### 2.6.2. TRAP Staining for Multinucleated Osteoclasts

At the end of culture, cells were fixed with 3.7 % formaldehyde in PBS and stained using a TRAP staining kit (#387A-KT, Sigma-Aldrich) according to the manufacturer's instructions. Large, TRAP-positive cells containing 3 or more nuclei were counted as mature osteoclasts [18]. Photomicrographs were taken with a Zeiss Discovery V20 Stereo Microscope equipped with an AxioCam MRc5 camera (Carl Zeiss AG).

#### 2.6.3. TRAP Activity in Conditioned Media

TRAP solution was prepared using a TRAP staining kit (#387A-KT, Sigma Aldrich) according to the manufacturer’s instructions. At the end of culture, TRAP solution was added to conditioned media (from the cells described in 2.6.2) and incubated at 37 °C for 3 hrs. TRAP activity was quantified by measuring optical absorbance at 550 nm using an Epoch Micro-plate spectrophotometer (BioTek).

#### 2.6.4. Bone Resorption Assay

For resorption assays cells were seeded into 24-well Corning osteoassay plates in the presence of 30 ng·mL^−1^ RANKL and increasing concentrations of honeybush tea extract for 5 days as previously described. At the end of culture, the cells were washed off with 5% sodium hypochlorite solution and a modified Von Kossa stain was used to stain the resorption pits. In brief, 5% silver nitrate in distilled water was added to each well and the plates were incubated in the dark for 30 min. Thereafter, the silver nitrate was replaced with 5% sodium carbonate in formalin for 5 min. The plate was then washed subsequently and left to dry. Photomicrographs of the resorbed areas were taken with a Zeiss Discovery V20 Stereo Microscope equipped with an AxioCam MRc5 camera (Carl Zeiss AG). The images were analyzed using ImageJ software [19].

#### 2.6.5. Visualisation of Multinucleated Cells with Actin Rings

At the end of culture, cells were fixed with 3.7% formaldehyde in PBS for 10 min. After fixation cells were permeabilized with 0.1% Triton X-100 for 10 min and stained with phalloidin conjugate solution (Sigma-Aldrich) for 40 min. Thereafter the cells were washed with PBS and the nuclei were stained with Hoechst 33342. Photomicrographs were taken using a Zeiss inverted Axiovert CFL40 microscope with a Zeiss Axiovert MRm monochrome camera (Carl Zeiss AG) using the appropriate filter sets: Hoechst (Excitation: 352 nm, Emission: 455 nm); Phalloidin (Excitation: 502 nm. Emission: 525 nm).

#### 2.6.6. Gene Expression Analysis

At the end of culture, total RNA was extracted using TRI Reagent® and reverse transcribed to cDNA using M-MuLV reverse transcriptase. Roche FastStart Essential DNA Green Master was used for the quantitative realtime PCR (qRT-PCR) assay. Analysis of relative gene expression was done using the 2^−ΔΔCT^ method and results were normalized to the housekeeping gene (GAPDH). All the primers used in the study were synthesized by Inqaba Biotec (Pretoria, South Africa) and are listed in Table 1.

**Table 1:** Primers used in this study.

Gene	Primer Sequences (5’ – 3’)	Genbank Accession Number
MMP9	GTCATCCAGTTTGGTGTCGCG AGGGGAAGACGCACAGCTC	NM_013599.3
TRAP	CCACCCTGAGATTTGTGGCT ACATACCAGGGGATGTTGCG	NM_008084.2
Cathepsin K	CTGGAGGGCCAACTCAAGA CCTCTGCATTTAGCTGCCTT	NM_007802.4
GAPDH	GATGACATCAAGAAGGTGGTGAAGC ATACCAGGAAATGAGCTTGACAAAG	NM_001102404.1

### 2.7. Statistical Analysis

The data represents results from three independent experiments conducted in triplicate, unless otherwise stated, and are represented as the mean ± standard deviation (SD). The data was analysed using one way analysis of variance (ANOVA) followed by Bonferroni *post hoc* test using GraphPad Prism software (GraphPad Software Inc., La Jolla, CA, USA). *p* < 0.05 was regarded as statistically significant.

## 3. Results

### 3.1. HPLC-DAD

HPLC analysis revealed the presence of the flavanone, hesperidin, the xanthones, mangiferin and isomangeferin, and the benzophenone, iriflophenone-3-C-glucoside (Table 2) which are all known to be present in honeybush tea. The presence of unknown compound 13 was notable as this compound is only known to be present in the *Cyclopia intermedia* species [17].

**Table 2:** Analysis of the honeybush tea extract for known flavonoid compounds. HPLC method was performed as previously published [17]. Concentrations are calculated averages from duplicate injections per sample (for the internal quality control, a %RSD below 5% was set to ensure accuracy) expressed as mg/L in undiluted tea extract.

Compound	Iriflophenone -3-C-Glucoside	Mangiferin	Isomangiferin	Hesperidin	Unknown 13
Concentration	9.665	10.009	9.952	6.426	6.092

### 3.2. Effect of Honeybush Tea Extract on Cell Viability

**Figure 1:**
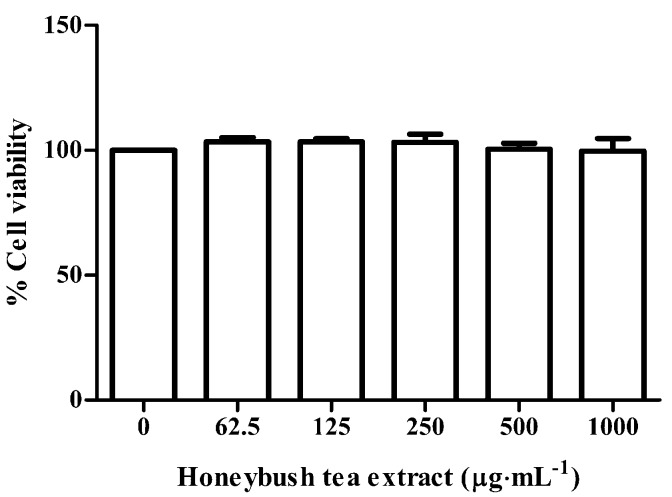
Effect of honeybush tea extract on cell viability. Cells were exposed to honeybush tea extract at increasing concentrations for 48 h. Data are expressed as the mean ±SD percentage relative to the control and are representative of three independent experiments conducted 6-fold.

To determine whether honeybush tea extract could have cytotoxic effects an Alamar blue assay was conducted. Cell viability was assessed at concentrations of 62.5–1000 µg·mL^−1^ of honeybush tea extract. As seen in Figure 1, these concentrations of honeybush tea extract showed no cytotoxic effects on the RAW264.7 murine macrophages and were therefore used for subsequent experiments.

### 3.3. Effect of Honeybush Tea Extract on Cell Morphology

PlasDIC is a microscopy technique that provides detailed, high quality images of cells in culture [20]. PlasDIC was used to observe morphological changes in RAW264.7 macrophages exposed to RANKL (15 ng·mL^−1^) alone or in combination with honeybush tea extract (62.5–1000 µg·mL^−1^).

**Figure 2:**
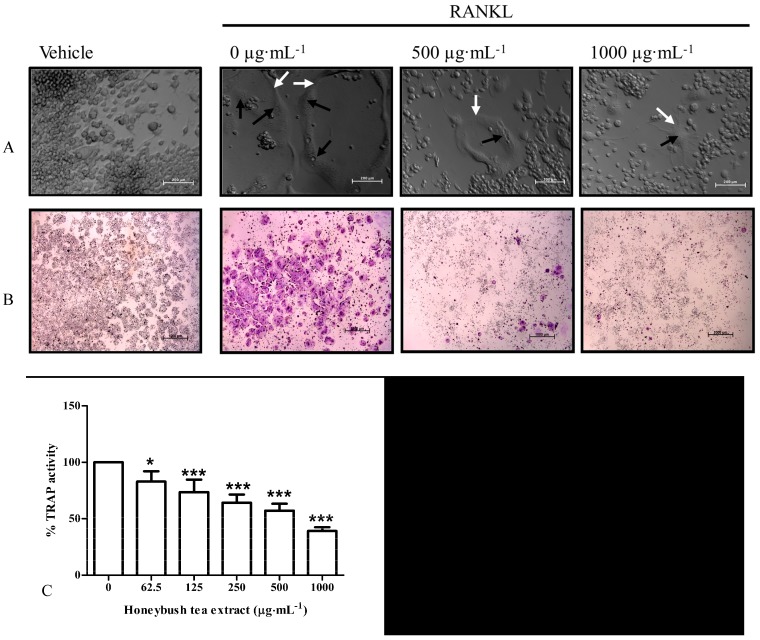
Effect of honeybush tea extract on cell morphology and osteoclast formation. (**A**) PlasDIC images. Osteoclast boundaries are indicated with white arrows while nuclei are indicated with black arrows. Scale bar = 200 µm. (**B**) Photomicrographs of osteoclasts in culture wells. Osteoclasts stain purple in the presence of TRAP. Scale bar = 1000 µm. (**C**) TRAP+ cells with three or more nuclei were counted as mature osteoclasts in each well. (**D**) TRAP activity was quantified and represented as a percentage relative to the control. Data are mean ±SD and are representative of three independent experiments conducted in triplicate. * *p* < 0.05; ** *p* < 0.01; *** *p* < 0.001 *vs.* control.

No differentiation was observed in cells exposed to vehicle alone. After exposure to RANKL, large, multinucleated osteoclasts were observed (Figure 2A). RANKL treated cells in combination with honeybush tea (62.5–1000 µg·mL^−1^) extract generated fewer osteoclasts compared to the control in a dose dependent manner. Higher concentrations of tea extract (500–1000 µg·mL^−1^) yielded morphologically smaller osteoclasts. No other distinctive morphological traits were observed.

### 3.4. Effect of Honeybush Tea Extract on RANKL-Induced Osteoclast Formation and TRAP Activity in RAW264.7 Macrophages

TRAP is an enzyme highly expressed and secreted by mature osteoclasts and is commonly used as a marker for osteoclast formation [4]. We sought to determine whether honeybush tea extract could affect the number of TRAP+ osteoclasts formed and TRAP activity. RAW264.7 macrophages were exposed to RANKL alone or in combination with honeybush tea extract (62.5–1000 µg·mL^−1^). RANKL induced the formation of osteoclasts. Large multinucleated cells with 3 or more nuclei, which stained purple for the presence of TRAP, were counted as mature osteoclasts (Figure 2B). Honeybush tea extract significantly reduced RANKL-induced osteoclast formation compared to the control (Figure 2C). Furthermore, honeybush tea extract significantly reduced TRAP activity in a dose dependent manner (Figure 2D).

### 3.5. Effect of Honeybush Tea Extract on Bone Resorption in RAW264.7 Macrophages

**Figure 3:**
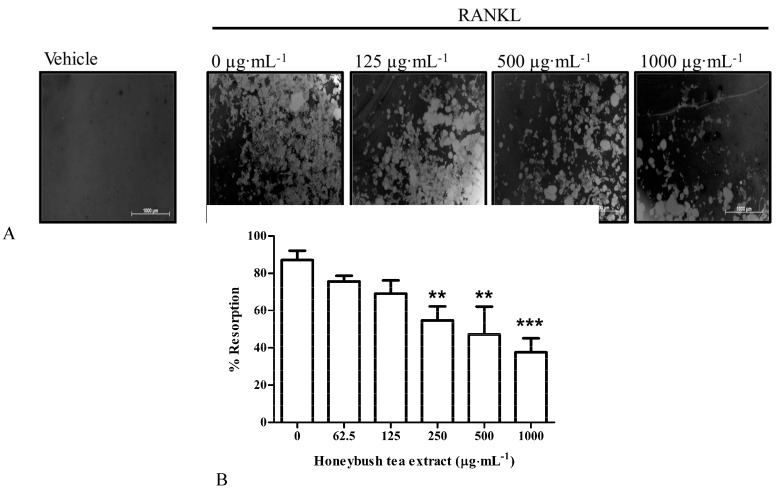
Effect of honeybush tea extract on bone resorption. Cells were seeded onto osteoassay plates and exposed to RANKL (30 ng·mL^−1^) alone or in combination with honeybush tea extract at increasing concentrations for 5 days. (**A**) Photomicrographs of resorption pit formation in 24-well osteoassay plates. Light areas are the resorbed surfaces. Scale bar = 1000 µm. (**B**) Resorption pit formation was quantified using Image J software. Data are mean ±SD and are representative of three independent experiments conducted in triplicate. * *p* < 0.05; ** *p* < 0.01; *** *p* < 0.001 *vs.* control.

The primary function of osteoclasts is to resorb the bone matrix [21]. We examined whether honeybush tea extract could affect resorption with the use of osteoassay plates. RAW264.7 macrophages were exposed to RANKL alone or in combination with honeybush tea extract (62.5–1000 µg·mL^−1^). RANKL-induced osteoclasts caused resorption on the osteoassay substrate (Figure 3A). Honeybush tea extract showed no significant effect on bone resorption at 62.5–125 µg·mL^−1^ compared to the control (Figure 3B). However, at concentrations greater than 250 µg·mL^−1^, the tea extract significantly reduced bone resorption concomitant to its effect seen on osteoclast formation.

### 3.6. Effect of Honeybush Tea Extract on Actin Ring Formation in RAW264.7 Macrophages

**Figure 4:**
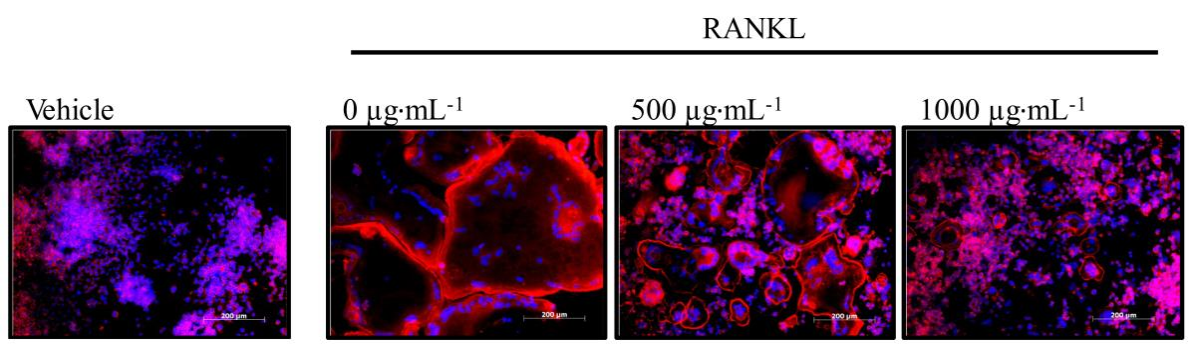
Effect of honeybush tea extract on actin ring formation. Cells were exposed to RANKL (15 ng·mL^−1^) alone or in combination with honeybush tea extract at increasing concentrations for 5 days. Osteoclasts were stained for actin with phalloidin (red) and for nuclei with Hoechst (blue). Scale bar = 200 µm.

The formation of the actin ring is a necessary feature for bone resorption to occur [21]. RAW264.7 macrophages were exposed to RANKL alone or in combination with honeybush tea extract (62.5–1000 µg·mL^−1^). Actin ring formation was visualized by fluorescent staining. As seen in Figure 4, large osteoclasts exhibited a red actin ring cytoskeletal structure that is characteristic of osteoclasts. Fewer and smaller actin rings were seen in cells exposed to honeybush tea extract at 500–1000 µg·mL^−1^ (Figure 4).

### 3.7. Effect of Honeybush Tea Extract on Osteoclast Specific Gene Expression in RAW264.7 Macrophages

**Figure 5:**
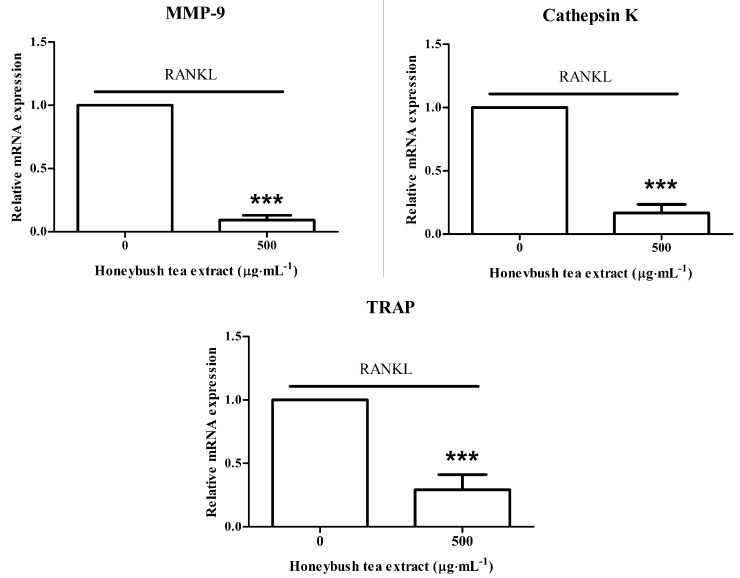
Effect of honeybush tea extract on osteoclast specific gene expression. Cells were exposed to RANKL (15 ng·mL^−1^) alone or in combination with honeybush tea extract at 500 µg·mL^−1^ for 5 days. Gene expression of osteoclastic markers, MMP-9, TRAP and cathepsin K, was determined by qRT-PCR. GAPDH served as the loading control. Data are mean ±SD and are representative of three independent experiments conducted in triplicate. * *p* < 0.05; ** *p* < 0.01; *** *p* < 0.001 *vs.* control.

The effects of honeybush tea extract were further characterized with respect to the expression of key osteoclast specific gene markers. RT-PCR was used to evaluate the expression of cathepsin K, TRAP and MMP-9. RAW264.7 macrophages were exposed to RANKL alone or in combination with honeybush tea extract at 500 µg·mL^−1^. Exposure to honeybush tea extract suppressed the expression of all the three key osteoclast specific markers compared to the control (Figure 5).

## 4. Discussion

Tea is one of the most widely consumed beverages in the world. Traditional teas (black, green, oolong, *etc.*) are made from the plant *Camellia sinensis* [22]. Herbal teas (honeybush, rooibos, *etc.*) are made from a variety of plant sources and are increasingly popular owing to their many reported health benefits [9]. Several studies have shown a positive association between tea drinking and bone mineral density [23,24,25,26]. However, potential bone protective effects of honeybush tea, a South African herbal tea known for its pleasant taste and aroma as well as its anti-inflammatory properties [9,10], remain unexplored. Certain chronic inflammatory disorders, such as arthritis and osteoporosis, are characterized by a decrease in bone formation by osteoblasts and an increase in bone resorption by osteoclasts [27]. Inhibition of osteoclast activity may help alleviate symptoms in patients suffering from these chronic inflammatory disorders. The present study examined the effect of aqueous extract of fermented honeybush tea on the formation and function of osteoclasts derived from RAW264.7 murine macrophages. To the best of our knowledge, this is the first study to evaluate the anti-osteoclastogenic potential of aqueous honeybush tea extract.

RAW264.7 macrophages express c-fms, the M-CSF receptor, as well as M-CSF and therefore do not require the addition of M-CSF in their differentiation into osteoclasts [28]. This cell line is also known to express high levels of RANK and is therefore suitable for investigating *in vitro* effects on RANKL-induced osteoclast formation [28]. Our results showed that aqueous honeybush tea extract inhibited RANKL-induced osteoclast formation in RAW264.7 macrophages. A number of polyphenolic compounds have been identified in aqueous extracts of honeybush tea [17,29]. Some of the major phenolic compounds found in honeybush teas include hesperidin, a flavanone, mangiferin and isomangiferin, which are xanthone C-glycosides, and the benzophenones, iriflophenone-3-C-glucoside-4-O-glucoside and iriflophenone-3-C-glucoside [30]. Hesperidin has been shown to inhibit bone loss in ovariectomized female mice [31] and androgen deficient male mice [32] while mangiferin has been shown to inhibit parathyroid-hormone stimulated bone resorption in mice [33]. We found that aqueous honeybush tea extract decreased the bone resorption potential of osteoclasts on osteoassay plates indicating a possible role of these polyphenols in the anti-osteoclastogenic effects of honeybush tea. Furthermore, Ang *et al.* have reported that mangiferin can inhibit RANKL-induced osteoclast formation, resorption and actin ring formation in mouse bone marrow macrophage (BMM) cultures [34]. The formation of the F-actin ring is necessary for bone resorption to occur and its appearance is a functional marker of osteoclasts [35]. We found that exposure to honeybush tea extract resulted in fewer and smaller actin rings compared to the control, possibly due to a decreased ability of osteoclasts to fuse resulting in fewer and smaller cells. However, Dudhia *et al.* have shown that the fermented honeybush tea, as used in this study, can decrease the polyphenolic content by 50% [36]. Interestingly, Dudhia *et al.* reported that the decrease in polyphenolic content did not inhibit the anti-adipogenic effects of honeybush tea extract, possibly indicating that the fermentation process of honeybush tea does not alter its effects [36]. These findings are of considerable importance as fermented honeybush tea is more popular than unfermented tea as it has a sweeter flavour. This study also reported that the fermented tea was less cytotoxic than unfermented tea in 3T3-L1 pre-adipocytes [36]. Similarly, we did not find effects of fermented honeybush tea extract on cell viability in RAW264.7 macrophages at the concentrations used in our study.

MMP-9, TRAP and cathepsin K are all key markers of osteoclasts that play an important role in resorption [2]. Cathepsin K is the most abundantly present cathepsin in the resorption lacunae and plays a crucial role in the degradation of the organic matrix as well as cleavage of TRAP into its activated form [37,38]. Disruption of the cathepsin K and TRAP gene have been shown to lead to osteopetrosis (a congenital disorder characterized by overly dense bones) [39,40]. MMP-9 also plays a role in the resorption of the organic matrix as elevated levels of this protein are present in the resorption lacunae [41]. Similar *in vitro* studies on green tea extract have shown an inhibitory effect of the extract on MMP-9 expression and osteoclast formation [42]. Interestingly, a further study observed that regular green tea drinkers had improved bone mineral density compared to non-tea drinkers [43].

We found that honeybush tea extract inhibited osteoclast formation and decreased MMP-9, TRAP and cathepsin K gene expression. Disruption of these crucial resorption genes may explain the anti-resorptive effect of honeybush tea extract observed in this study. Hence, our study demonstrates that commercial honeybush tea extract may have potential beneficial effects on bone, possibly through the synergistic action of its bioactive compounds.

## 5. Conclusions

In conclusion we found that commercial honeybush tea extract can inhibit RANKL-induced osteoclast formation and bone resorption in RAW264.7 murine macrophages. This study, for the first time, demonstrates anti-osteoclastogenic effects of honeybush tea which may indicate potential bone protective effects.
